# Novel Biorecognition Elements against Pathogens in the Design of State-of-the-Art Diagnostics

**DOI:** 10.3390/bios11110418

**Published:** 2021-10-26

**Authors:** Maria G. Sande, Joana L. Rodrigues, Débora Ferreira, Carla J. Silva, Ligia R. Rodrigues

**Affiliations:** 1 CEB—Centre of Biological Engineering, Campus de Gualtar, Universidade do Minho, 4710-057 Braga, Portugal; msande@centi.pt (M.G.S.); joanarodrigues@ceb.uminho.pt (J.L.R.); deboraferreira@ceb.uminho.pt (D.F.); 2 CENTI—Center for Nanotechnology and Smart Materials, Rua Fernando Mesquita 2785, 4760-034 Vila Nova de Famalicão, Portugal; cjsilva@citeve.pt; 3 CITEVE—Technological Center for the Textile and Clothing Industries of Portugal, Rua Fernando Mesquita 2785, 4760-034 Vila Nova de Famalicão, Portugal

**Keywords:** biorecognition, diagnosis, biosensor, pathogens, aptamers, antibodies, peptides, enzymes, DNAzymes, peptide nucleic acids

## Abstract

Infectious agents, especially bacteria and viruses, account for a vast number of hospitalisations and mortality worldwide. Providing effective and timely diagnostics for the multiplicity of infectious diseases is challenging. Conventional diagnostic solutions, although technologically advanced, are highly complex and often inaccessible in resource-limited settings. An alternative strategy involves convenient rapid diagnostics which can be easily administered at the point-of-care (POC) and at low cost without sacrificing reliability. Biosensors and other rapid POC diagnostic tools which require biorecognition elements to precisely identify the causative pathogen are being developed. The effectiveness of these devices is highly dependent on their biorecognition capabilities. Naturally occurring biorecognition elements include antibodies, bacteriophages and enzymes. Recently, modified molecules such as DNAzymes, peptide nucleic acids and molecules which suffer a selective screening like aptamers and peptides are gaining interest for their biorecognition capabilities and other advantages over purely natural ones, such as robustness and lower production costs. Antimicrobials with a broad-spectrum activity against pathogens, such as antibiotics, are also used in dual diagnostic and therapeutic strategies. Other successful pathogen identification strategies use chemical ligands, molecularly imprinted polymers and Clustered Regularly Interspaced Short Palindromic Repeats-associated nuclease. Herein, the latest developments regarding biorecognition elements and strategies to use them in the design of new biosensors for pathogens detection are reviewed.

## 1. Introduction

Infectious diseases remain a significant global health concern and cause of mortality. Lower-respiratory infections, diarrhoeal diseases and tuberculosis are currently among the top ten global causes of mortality [1]. A worrying development that will greatly hamper treatment and control of infectious diseases is the emergence of drug-resistant pathogens in recent decades. Currently, at least 700,000 people around the world die each year due to infections caused by drug-resistant organisms [2].

For centuries, clinical manifestations were the most common means to establish a diagnosis for many infections. Remarkably, this is still often the case today because diagnosis of an infection can take several days before a result is delivered. The classical methods for microbial identification are based on culture, which is a slow process providing low sensitivity and has limited use in viral identification. Other traditional microbial diagnostics use microscopy and staining, and serological methods, such as hemagglutination assays. Although inexpensive and rapidly performed, these methods suffer from the disadvantages associated with culturing [3,4]. More recently, standard molecular methods have been adopted for microbial diagnosis, such as Polymerase chain reaction (PCR) and Matrix-assisted laser desorption/ionisation time-of-flight mass spectrometry (MALDI-TOF). These methods, although highly sensitive and relatively fast to perform, are complex and require expensive sophisticated equipment with low portability, often making them inaccessible [3].

To successfully manage infectious diseases in a clinical setting, a rapid diagnosis and administration of targeted antimicrobial or antiviral therapy is crucial [5]. Because current solutions can fail to quickly identify serious infections, they can prove fatal due to a delay in treatment. Examples of such conditions include sepsis, a potentially life-threatening condition [6], and *Clostridium botulinum* infection, which produces lethal neurotoxins that can lead to rapid deterioration of a patient but is sometimes misdiagnosed as stroke or other conditions. To improve diagnostic outcomes, the World Health Organization (WHO) defined that the ideal diagnostic tests should be affordable, sensitive, specific, rapid, equipment-free and delivered to those who need them [7]. Most conventional methods do not meet all or most of these criteria. With the aim of meeting these criteria, point-of-care (POC) diagnostics such as biosensors for mass application are under development because they are inherently flexible, easy to use, minimally instrumented and can be produced at low costs [8].

Biorecognition elements, also referred to as bioreceptors, have the essential function of providing analyte specificity to a biosensor. An ideal biorecognition element possesses a selective and potent affinity towards the bioanalyte, thus endowing a biosensor with good specificity [9]. This review provides an update on the different types of biorecognition elements being studied and the strategies employed for the identification of pathogens that can be used for POC diagnostics. Although the use of antibodies for biorecognition dominates the landscape due to their inherent abilities for antigen detection, there are a few challenges associated with their use in biosensors, such as instability and high cost. Hence, recent research has been more focused on nucleic acid derivatives such as aptamers and peptide nucleic acids (PNAs), which can be modified to improve their biorecognition capabilities while being arguably more stable than antibodies. Numerous studies have been conducted on aptamers and the most recent ones are herein summarised. In addition, theranostic approaches which combine therapy and diagnosis [10] make use of the dual properties of antibiotics and certain types of peptides to selectively detect and simultaneously inactivate pathogens, making them extremely useful, and several examples are discussed. Enzymes, similarly to antibodies, have intrinsic properties for pathogen identification and have also been adapted to some extent, although they also suffer from instability. Other recent strategies for pathogen identification are also explored, such as Clustered regularly interspaced short palindromic repeats/associated nuclease (CRISPR-Cas) and molecularly imprinted polymers (MIPs).

This plethora of promising biorecognition elements are versatile enough to be used by themselves, routinely coated onto magnetic nanoparticles (MNPs), microparticles and microfluidic devices or included in detection probes where they are integrated into biosensors for pathogen identification. All these strategies and methods are thoroughly discussed in this review with a focus on sensing platforms used especially in POC diagnostics. Although most of the reviews related to biosensors are more focused on the detection strategies and principles [11,12], our review addresses the important aspect of how exactly the pathogenic targets are identified or recognised prior to detection. In addition, a number of examples of commercially available biosensors are examined. This aspect is often overlooked in other reviews, which usually only consider results from research outputs.

## 2. Recent Progress in Platforms for Pathogen Detection

**Figure 1:**
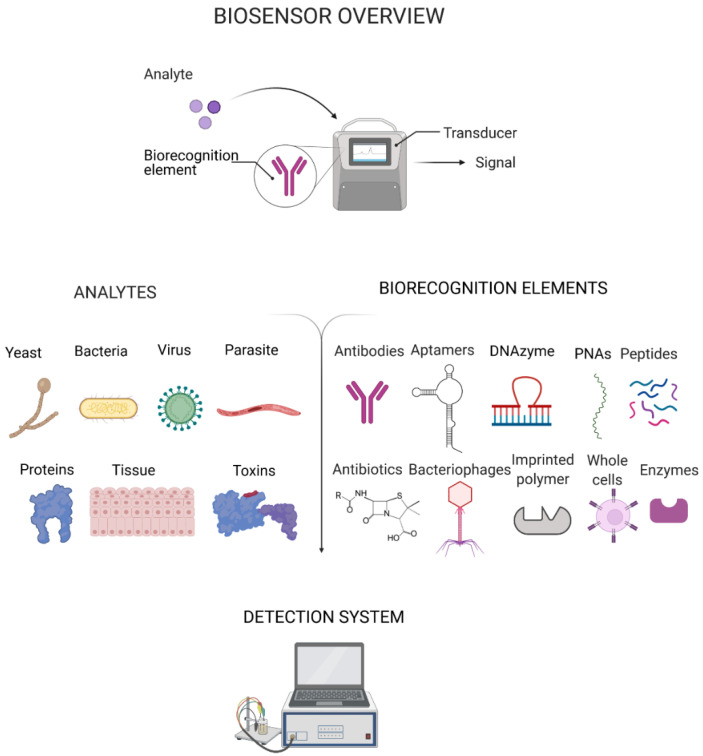
Main components of a biosensor: analyte, biorecognition element and transducer which produces a detectable signal. The major types of biorecognition elements and analytes in the context of clinical pathogen detection are included. PNAs: peptide nucleic acids. Created with BioRender.com.

In the past few decades, biosensors have emerged as an alternative to conventional diagnostic methods. Biosensors are highly advantageous due to their high sensitivity, low-cost, ease-of-use and rapid response time for analyte detection [12]. A biosensor comprises a biorecognition element that binds to a target analyte (e.g. bacteria, virus, protein, polysaccharide) and transducer elements that translate the binding occurrence into a detectable signal with a detector providing readout (Figure 1) [13]. The translation of prototype biosensors to commercial development has hitherto been a slow process. However, in response to the emerging need for POC diagnostics and the manifold benefits of sensor-based testing, numerous new commercial biosensors are being introduced to worldwide markets.

Table 1 highlights some sensor-based products for the identification of pathogens that are currently available on the market. Most of these products are based on immunological principles using antibodies to recognise the analyte. These commercial sensor platforms are usually in the form of lateral flow immunoassays (LFA), latex agglutination assays and rapid antigen tests. Other classes of novel biorecognition elements that have shown some promise in pathogen identification are still not commonly used in commercial sensors. Regarding the detection systems, biosensors based on electrochemical, piezoelectric and optical principles are still not widely commercialised. 

Other advancements to improve diagnostics for infectious diseases make use of computational methods and tools. More recently, computational modelling has been used to design biorecognition elements with an effectiveness unlikely to be matched by conventional wet laboratory techniques. Molecular docking is an example of a computational modelling technique that has been used to design high-affinity biorecognition elements towards the detection of pathogenic targets [34]. For ligand discovery, in silico simulations have been used to design “superselective” multivalent probes by optimising the multiplicity (binding to the target DNA at multiple sites), instead of the strength of the probe-target bond, wherein the target can be pathogens, to enhance detection sensitivity and specificity [35]. Technologies such as Next-Generation Sequencing (NGS) or massively parallel sequencing, which are high throughput sequencing methods, have the potential to establish hypothesis-free diagnostic approaches to detect virtually any pathogen leading to a paradigm shift in how infections can be diagnosed [3]. Metagenomic NGS (mNGS) has proven capabilities of detecting a wide range of pathogens present in a patient sample [36]. Additionally, the enrichment of target nucleic acids prior to mNGS enhances the sensitivity of detection [37]. To illustrate, Wylie and collaborators [38] developed a capture probe called ViroCap, constituted by a panel of 2 million sequences able to enrich viral nucleic acids of a large set of eukaryotic viruses preceding mNGS to increase the sensitivity of virus detection. All the expected viruses from 26 clinical samples and 30 additional viruses were detected, whereas when only mNGS was used some of the viruses were not detected. Although mNGS can be successfully implemented, it is time- and resource-intensive and requires bioinformatics expertise. Therefore, its potential in diagnostics is based on specific cases where unsuspected or unculturable organisms are present and may be not detected (false negative results) with standard assays. 

## 3. Biorecognition Elements

The essential requirement of an effective biorecognition element is to provide specificity for the (bio)analyte (also referred to as the target) [9]. The most important characteristics of a biorecognition element for pathogen identification are sensitivity (few false negatives) and selectivity (few false positives) [39]. Widely used as biorecognition elements are the antibodies’ immunoglobulins (Ig) due to their exceptionally high affinity and specificity towards their target analytes. Moreover, aptamers, peptides, bacteriophages and enzymes, among others, have also been established as being effective for biorecognition purposes. The targets of biorecognition elements, which enable pathogen identification, are usually specific surface molecules (e.g., biomarkers), such as proteins and epitopes or their by-products (toxins and metabolites) or nucleic acids. Exploring the various biorecognition elements and their inherent characteristics is essential to improve and develop novel POC diagnostics.

### 3.1. Antibodies

Igs, generally referred to as antibodies, are large glycoproteins produced by white blood cells with strong affinity and specificity towards their target analytes. These qualities make them a natural and popular choice as biorecognition elements and consequently, have been adapted for use in pathogen identification. In addition to whole monoclonal antibodies (mAbs), which are laboratory made, antibody-derived single-chain variable fragments (scFv) and fragment antigen-binding (Fab’) units are commonly used for biorecognition [40]. They are more cost-effective than mAbs while providing similar specificity values as conventional antibody approaches. Antibody-based probes are most commonly used for the detection of specific proteins and whole cells [41]. Because numerous methods of pathogen identification using antibodies as biorecognition elements have been reported, this review provides only a brief overview of a few recent reported strategies. 

Antibodies are identified within the relevant biochemical pathways and then produced in animals and purified for various applications, including for use as capture probes in sensors platforms [9]. Most examples of antibody-based biosensors for detection of pathogens use commercially produced antibodies, as in the case of Park et al. [42] who developed a 3D printed microfluidic device with antibodies conjugated to MNPs (Ab-MNPs) as capture probes against the common pathogen enterohaemorrhagic *Escherichia coli* O157:H7. The device, termed a magnetic pre-concentrator (3DμFMP) was tested using human blood mixed with 10^2^–10^7^ colony forming units (CFU) of *E. coli* O157:H7 and Ab-MNPs (10^13^ particles/mL). The mixture was treated with adenosine triphosphate (ATP) eliminating reagent before being injected into the 3DμFMP device. Captured cells were then magnetically separated and quantified using an ATP luminescence assay achieving an excellent limit of detection (LOD) of 10 CFU/mL in blood. The 3DμFMP device was efficient, selectively enriching *E. coli* O157:H7 700-fold in a volume of 100 mL in one hour.

**Figure 2:**
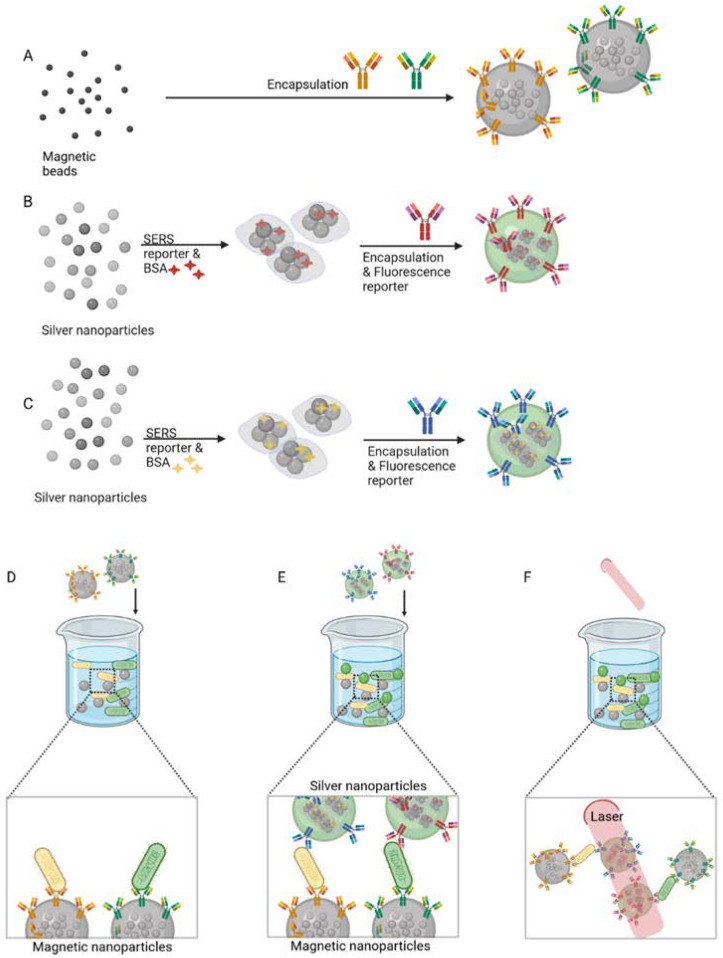
Three-step synthesis of monoclonal antibodies (mABs) conjugated with magnetic beads and SERS (surface-enhanced Raman spectroscopy) and fluorescence-based dual nanoprobes for the multiplex detection of *Escherichia coli* J5 and *Francisella tularensis*: (**A**) Magnetic bead clusters were encapsulated and conjugated to mABs to selectively capture either *E. coli* or *F. tularensis*. (**B**,**C**) For the subsequent detection step after bacterial capture, AgNP clusters were encoded with SERS reporters (red and yellow stars), stabilised by bovine serum albumin (BSA), conjugated further with fluorescent dyes and encapsulated in a polymer. (**D**) *E. coli* J5 and *F. tularensis* bind to the respective mABs and were magnetically separated. (**E**,**F**) Multiplex detection of sandwich immunocomplexes composed of bacteria, magnetic bead clusters and the SERS and fluorescence-based dual nanoprobes was achieved. Detection method develop by Jang et al. [43]. Created with BioRender.com.

Antibodies were also used in multiplex detection of pathogenic bacteria by Jang et al. [43]. Figure 2 illustrates the synthesis of modified capture magnetic beads (MBs) and dual nanoprobes for detection, which are based on both surface-enhanced Raman spectroscopy (SERS) and fluorescence. Two different mAbs that recognise two pathogens, *E. coli* J5 and *Francisella tularensis,* were conjugated to respective MB-clusters for selective magnetic capture (Figure 2A). The dual nanoprobes used for detection (Figure 2B,C), comprised silver nanoparticles (AgNPs) conjugated with SERS reporters and fluorescence dyes and, subsequently, the AgNP clusters were encapsulated. They were conjugated to another pair of mAbs that recognise the respective bacterial targets (Figure 2A). In the presence of *F. tularensis* and *E. coli* J5, the MBs-clusters selectively bind to the respective bacterial target (Figure 2D). Following magnetic enrichment, the dual nanoprobes were added, which formed sandwich-type immunocomplexes containing the bacteria, MB-clusters and the dual nanoprobes (Figure 2E). Detection was performed by SERS (Figure 2F) and fluorescence microscopy. A linear correlation was verified between the Raman intensity and the pathogen concentration from 10^2^ to 10^6^ cells/mL, and the LOD was found to be less than 10^2^ cells/mL. 

Another similar example adapted the use of antibody conjugated NP enrichment with SERS detection in an easy-to-use LFA format. Primary antibody-conjugated magnetic gold nanoparticles (AuNPs) were used for enrichment of *E. coli.* The enzyme rennet was used to prevent aggregation of the AuNPs, to facilitate free movement of the bacteria along the paper-based LFA strip followed by SERS detection. The sensitivity of this system was comparable with a plate-counting method and could be completed in 1 h [44].

A POC biosensor for the identification of *Mycobacterium tuberculosis* was reported using monoclonal antibodies with high specificity towards the well-established *M. tuberculosis* heat shock protein X (HspX) [45]. The antibodies were directly immobilised on a plasmonic sensor surface for detection, achieving a LOD of 0.63 ng/mL in pretreated sputum samples. Using a similar strategy, Zika virus was detected by immobilising a monoclonal antibody specific to an envelope protein of Zika virus onto an impedimetric immunosensor [46]. The sensor achieved a low LOD of 10 picomolar.

Although antibodies have been the gold standard as affinity molecules for decades, their limitations include poor stability and reproducibility, which complicate their use in sensing platforms that require a long shelf-life. Other problems relate to lengthy production time and the need for ethical approval, which increase costs [47]. Therefore, research in recent years has also focused on finding alternate biorecognition elements with improved specifications.

### 3.2. Enzymes

Enzymes are natural, biologically derived molecules that have evolved an innate ability to achieve analyte specificity [9]. Enzymes attain specificity to a bioanalyte through binding cavities deep in their structure. Binding can occur by various means such as hydrogen-bonding, electrostatic binding and other non-covalent interactions [48]. However, enzymes, such as antibodies, are sensitive to degradation, which affects their reproducibility and, by extension, their applicability in biosensors. Liu et al. [49] developed a microwave-assisted method for the synthesis of red fluorescence gold nanoclusters (AuNCs) functionalised with lysozyme and demonstrated their potential use in a visual detection nanosensor of *E. coli*. Lysozyme is advantageous as it reduces Au^+^ ions and thus stabilises the AuNCs. Additionally, lysozymes are antimicrobial enzymes that can recognise and kill several types of bacteria [50]. Due to lysozyme’s high specificity for *E. coli* and the fluorescence enhancement of the lysozyme functionalised AuNCs, the nanosensor detected *E. coli* with good sensitivity (LOD = 2 × 10^4^ CFU/mL). Due to the nature of the fluorescence evolution, the nanosensor can be suitably applied to real-time and fast detection of bacteria.

Label-free and real-time genus-specific detection of bacterial pathogens using lytic enzymes was proposed by Couniot et al. [51]. The lytic enzyme lysostaphin selectively digests staphylococci. Therefore, a compatible microelectrode sensor with *Staphylococcus epidermidis* anchored to its surface was incubated with lysostaphin. A real-time shift in impedance was observed when target bacteria were lysed by the enzyme. The LOD was calculated as 10^8^ CFU/mL within a minute of bacterial incubation. Specificity of the lysostaphin-based sensor to *S. epidermidis* was demonstrated in synthetic urine also containing *Enterococcus faecium*.

More recently, Clemente et al. [52] proposed a simple visualised method for rapid diagnosis of the respiratory pathogen *Pseudomonas aeruginosa* due to enzymatic liquefaction of infected sputum samples. The catalytic conversion of hydrogen peroxide, when added to an infected sample, by the *P. aeruginosa* enzyme catalase results in disruption of biofilms and generates an effervescence of oxygen bubbles which can be clearly seen. Catalase is produced only by *P. aeruginosa* and so other bacteria do not produce a reaction on addition of hydrogen peroxidase. An LOD of 10^5^ cells/mL was reported, which is considered the clinical threshold for respiratory infections detection. An important advantage of using enzymes instead of affinity molecules is that non-specific binding on sensors is eliminated, which could greatly facilitate its use for real samples. Moreover, lytic enzymes can be potentially applied in the detection of all Gram-positive bacteria.

### 3.3. Peptides

Peptides typically consist of short chains of 10–40 residues [53]. Antimicrobial peptides (AMPs) are part of the innate immune system in many eukaryotes. They usually bind to the negatively charged cell membranes of bacteria prompting cell lysis. AMPs have been successfully applied as biorecognition elements, in part due to their broad-spectrum activity against a variety of pathogenic bacteria. Cell-penetrating peptides (CPPs) are another class of short peptide sequences that find application as biomolecular delivery vehicles due to their ability to breach cellular membranes [54]. Both AMPs and CPPs have potential as biosensors and therapeutics due to their ability to inactivate pathogens [55]. Synthetic peptides can be selected randomly by phage display from phage libraries for application in biosensors and often perform better than antibodies [53,54]. Their relatively easy synthesis and intrinsic stability render them as suitable candidates to increase the shelf-life of sensor diagnostic platforms [56]. However, some key challenges are related to detection of bacteria in real samples, and their relatively low sensitivity and selectivity for diagnostic applications remain [53,57].

Certain pathogens can be identified indirectly based on their distinct by-products. For example, *E. coli* and some other bacterial species produce high levels of alkaline phosphatase (ALP). Zhang et al. [58] constructed a fluorescent probe that senses bacterial ALP activity. The novel probe comprised a controlled aggregation-induced emission luminogen (AIEgen) conjugated with a self-assembling peptide. The AIEgen-peptide probe designated as TPEPy-^D^F^D^F_P_Y^D^EG^D^K (TPEPy-pY) was designed containing a phosphorylated tyrosine, which, in the presence of bacterial ALP, is dephosphorylated. The dephosphorylation of TPEPy-pY reduces its hydrophilicity, causing the probe to assemble on the bacterial surface, triggering the AIEgen to fluoresce. The probe demonstrated good sensitivity and selectivity for ALP activity, with an LOD of 3.38 × 10^6^ CFU/mL.

Numerous examples of biorecognition methods make use of AMPs. In a recent study, Yuan et al. [59], reported MNPs modified with an AMP bacitracin A were able to capture bacteria. Their work revealed that the interactions between bacitracin A and bacteria are due to a pyrophosphate group present in the lipid target on bacteria and other indirect interactions mediated by sodium and zinc ions. After magnetic separation, SERS tags bound to the captured bacteria were used for their detection. The SERS spectra allowed to distinguish between *E. coli, Staphylococcus aureus* and *P. aeruginosa*.

Seminal work related to AMPs employed Magainin I to semi-selectively identify pathogenic bacteria [56]. Magainin I (GIGKFLHSAGKFGKAFVGEIMKS), which is naturally present on the skin of African clawed frogs, exhibits antibiotic activity against numerous species of bacteria. A micro-capacitive electrode sensor was functionalised with Magainin I. The sensor demonstrated adequate selectivity to distinguish strains of specific pathogenic Gram-negative bacteria, while Magainin I retained broadband detection abilities. A sensitivity to *E. coli* of 1 bacterium/μL was achieved. Another enrichment and detection platform comprised of a chemically modified microfluidic platform immobilised with Magainin I to capture bacteria achieved an LOD of 5 CFU/mL of *Salmonella* and 10 CFU/mL of *Brucella* from urine samples within 60 min [60].

**Figure 3:**
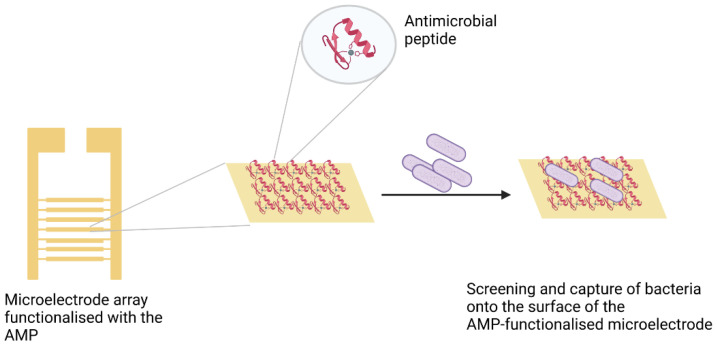
Graphical representation of an antimicrobial peptide (AMP)-based biosensor used for impedimetric detection of bacteria. The AMP is immobilised on a microelectrode array. The functionalised sensor selectively captures the target cells due to the immobilised AMP. Created with BioRender.com.

Class IIa bacteriocin AMPs such as leucocin A are noted for their anti-*Listeria* activity. A study by Azmi et al. [61] highlights these attributes of leucocin A. A peptide array was used to screen short peptides from a synthetic peptide library with selectivity for *Listeria monocytogenes* for use as biorecognition elements in a sensor. By employing this screening method, the Leucocin A fragment Leu10 (GEAFSAGVHRLANG) exhibited the highest affinity to target bacteria relative to the other peptide fragments. Similarly, leucocin A was used to selectively capture *L. monocytogenes* from among other Gram-positive strains followed by impedimetric detection, as illustrated in Figure 3. An LOD of 10^3^ CFU/mL was achieved by this method [62]. 

In some cases AMPs perform better than antibodies, as illustrated by Arcidiacono et al. [63], who evaluated the potential of fluorescently labelled AMPs as an alternative to labelled antibodies in the detection of *E. coli* O157:H7. AMPs cecropin P1, SMAP29 and PGQ were labelled with a fluorescent dye Cy5 and screened using a cell binding assay. It was revealed that Cy5-cecropin P1 improved the detection of target bacteria 10-fold when compared to a Cy5 labelled with an anti-*E. coli* O157:H7 antibody.

Other miscellaneous AMPs that have been researched for their biorecognition capabilities include Clavanin A and Ubiquicidin. Clavanin A is isolated from the marine tunicate *Styela clava*. An electrochemical biosensor was constructed using AuNPs chemically modified with cysteine and functionalised with Clavanin A for the detection of *Salmonella typhimurium* and *E. coli*. The sensor displayed moderate sensitivity and was able to differentiate signals for bacterial concentrations between 10^1^ and 10^4^ CFU/mL [64]. In a different study, a chemically modified version of Ubiquicidin enhanced with a fluorophore for detection by optical endomicroscopy demonstrated selectivity for pathogenic bacteria, and for the pathogenic fungus *Aspergillus fumigatus* (that causes pulmonary infections) over human cells in an ex vivo human lung model [65].

Multiplex systems based on arrays of various AMPs can be used for biorecognition of various analytes such as viruses. Fluorescently labelled viruses can be identified based on their characteristic response pattern upon interaction with the AMP panel [66]. Similarly, the goal of a study conducted by Kulagina et al. [67] was to establish such a multiplex detection system for pathogenic bacteria and viruses relevant to biodefence. The authors developed an array of different types of AMPs immobilised on a sensing substrate. The pathogens were fluorescently labelled and their identification was based on an evaluation of their binding pattern to the immobilised AMPs. The pathogens tested were Venezuelan equine encephalitis virus (VEE), vaccinia virus, *Brucella melitensis* and *Coxiella burnetii*. The AMPs array comprised polymyxins B and E, cecropins A, B, and P, melittin, parasin, bactenecin and Magainin I, and antibodies were used as controls. After the binding assays, it was observed that most of the immobilised AMPs bound labelled vaccinia virus, VEE and *C. burnetii* proportional to their concentration, and *B. melitensis* bound to bactenecin, polymyxin B and E. By establishing the characteristic binding patterns of various microbial pathogens, AMP arrays can be used to distinguish between them.

In a recent example, Fu et al. [68], developed a sensor array comprised of fluorogenic peptide probes for the differential sensing of the Ebola virus (EBV). The probes were constructed based on self-assembly between graphene oxide, which is a strong fluorescence quencher, and three fluorescently labelled peptide fragments T-RS5, T-QY7 and T-ED17, which were derived from antibodies. In the presence of pseudo-viruses (not able to replicate), the probes displayed an increase in fluorescence proportional to virus concentrations. This suggests that the peptide probes are removed from the graphene oxide surface to form peptide-virus complexes, resulting in fluorescence recovery. Based on the analysis of the fluorescence signals, differential sensing is evident in spite of the similarity of the viral capsid glycoproteins of EBV, Marburg virus and vesicular stomatitis virus.

A peptide microarray approach has also been developed to improve the accuracy of COVID-19 diagnosis. For example, Li et al. [69] prepared a microarray of spike protein (S1)-derived peptides from SARS-CoV-2 with full S1 coverage and analysed the immunological response from 2434 serum samples of COVID-19 patients including asymptotic patients. Based on the results, several 12-mer peptides were identified as suitable antigens to detect antibodies against SARS-CoV-2. While monitoring the IgG response, one of the peptides exhibited a sensitivity of 95.5% and specificity of 96.7%, which is comparable to the performance of the S1 itself for detection of symptomatic and asymptomatic COVID-19 cases. Additionally, a panel of four selected peptides was constructed with capabilities to prevent potential cross-reactivity with serum containing other coronaviruses. Additionally, Cai et al. [70] also evaluated a serological method for COVID-19 diagnosis and developed a peptide-based magnetic chemiluminescence enzyme immunoassay (MCLIA). Twenty candidate peptide epitopes of antigens of SARS-CoV-2 were predicted in silico and synthesised. The peptides were then linked to MBs and tested in an MCLIA with serum from COVID-19 patients to detect their binding to IgG and IgM antibodies. A peptide derived from the S1 protein of the virus showed the best performance and was further evaluated in an MCLIA with serum from 276 patients with COVID-19. The IgG and IgM positive detection rates were found to be 71.4% and 57.2%, respectively. By comparison, Pomplun et al. [71] screened peptides with high affinity towards the receptor-binding domain (RBD) of SARS-CoV-2 S1 towards the development of an efficient SARS-CoV-2 diagnostics. A method based on affinity selection using mass spectrometry (MS) was used to rapidly screen a library composed of 800 million synthetic peptides. Three sequences were identified with dissociation constants in the range 80–970 nM for the RBD. It was also shown that the RBD was selectively enriched by the selected peptides from a complex matrix comprising human serum proteins.

Because numerous peptides have been successfully tested for their biorecognition capabilities in sensing platforms and in arrays, they are promising candidates for use in biosensors, especially when taken together with their various other stated benefits.

### 3.4. Nucleic Acid Derivatives

The development of in vitro selection methodologies, such as the so-called “Systematic evolution of ligands by EXponential enrichment” (SELEX), to screen for single stranded DNA or RNA (ssDNA or ssRNA) from random-sequence nucleic acid libraries with high-affinity binding properties gave rise to intensive investigation on synthetic nucleic acids with special properties [72]. Since then, a variety of functional synthetic nucleic acids and their analogues have been developed for various diagnostics and therapeutic applications, including aptamers, DNAzymes and PNAs [73,74]. These synthetic molecules have distinct advantages over traditional antibodies as they are arguably more stable, versatile and cheaper to produce, making them the current preferred choice across sensing platforms [47].

#### 3.4.1. Aptamers

Aptamers are short ssDNA or ssRNA molecules, having a length of 25–100 bases, that fold into stable three-dimensional conformations. Due to their structure they recognise and bind to targets via hydrogen bonding, van der Waals forces and/or electrostatic interactions [54,75,76]. This behaviour makes them ideal biorecognition elements for targeting pathogens with high affinity and specificity. Compared to antibodies, aptamers exhibit significant advantages, including lower molecular weight, easier and cheaper production methods and good chemical stability [77,78,79].

**Figure 4:**
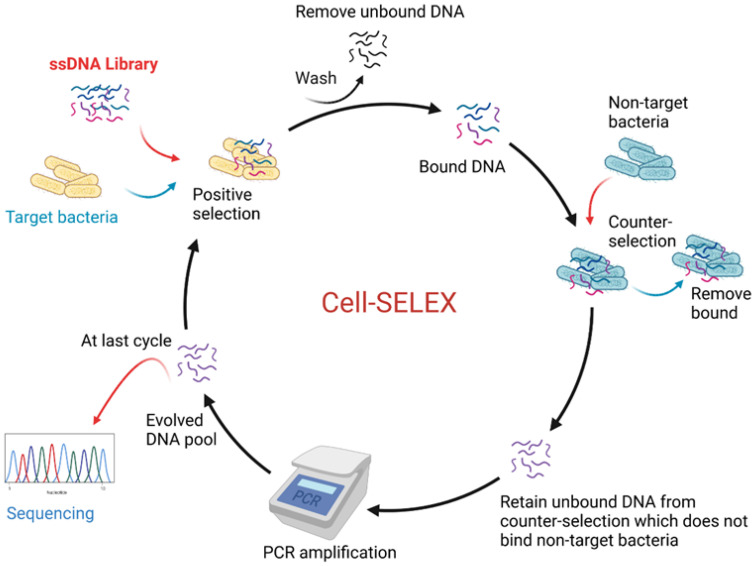
The general steps involved in the selection of candidate aptamers towards a target bacterium by cell SELEX (systematic evolution of ligands by exponential enrichment). These include incubation of the target bacteria with a single stranded DNA (ssDNA) library, washing steps, counter-selection and PCR (polymerase chain reaction) amplification before the cycle is repeated. After all cycles are completed, candidate aptamers are sequenced. Created with BioRender.com.

In addition, another advantage of aptamers is that they can be raised against an extensive variety of targets from small molecules to big proteins and also live cells, such as whole bacteria, for example [80]. The general steps of SELEX are illustrated in Figure 4. They can be briefly described as: (1) the target molecules (or cells) are incubated for a defined period with a ssDNA library pool; (2) non-binding sequences are then rinsed off; (3) next, bound sequences are recovered and PCR-amplified—for example, by using fluorescein isothiocyanate-labelled sense primers and biotin-labelled antisense primers; (4) finally, the antisense strands are removed to generate ssDNA pools for subsequent cycles of selection. As the cycles progress, various parameters, such as incrementing the number rinses, can be performed to increase stringency, allowing retention of aptamers with the strongest affinity from the pool. Flow cytometry is often used to monitor the enrichment of the selected pools by binding assays with the target, whereby selected pools with increased fluorescence are compared to the DNA library. To increase specificity to the target, the recovered pools obtained after some of the rounds are incubated with control molecules/cells to filter out sequences that bind to common sites present on the target, in addition to on the control [81,82].

The selected aptamers are being used as biorecognition elements in sensing platforms to detect a wide variety of pathogens in patient samples. For example, an innovative approach was developed by Wang et al. [75] to produce an aptamer-based device for application in bacterial infection diagnostics. The authors screened aptamers using cell-SELEX and identified highly specific aptamers capable of recognising three nosocomial and antibiotic-resistant bacteria, namely *E. coli*, *Acinetobacter baumannii* and the multidrug-resistant *S. aureus.* These aptamers were further integrated into a microfluidic system to form a paper-based dual-aptamer microfluidic chip. Compared with traditional laboratory techniques, this microfluidic system exhibited many advantages, including faster detection times, smaller size, higher specificity and multiplex capabilities.

In another study, Savory and collaborators [83] used bacterial cell-SELEX to screen for aptamers against *Proteus mirabilis*, which causes catheter-associated urinary tract infections, in combination with in silico maturation (ISM) to improve aptamer specificity. ISM uses a genetic algorithm to predict aptamer sequences with stronger target affinity than the promising parent sequences raised by cell-SELEX. This is achieved through successive rounds of sequence scrambling and random mutation in silico, followed by functional screening in vitro and selection of improved aptamers. After two cycles of ISM, one aptamer displayed a 36% higher specificity value than the original sequence selected by cell-SELEX [83].

Aptamers also find use in the detection of tuberculosis [84]. During the early stages of infection by virulent *M. tuberculosis*, culture filtrate protein 10 (CFP10) and early secreted antigen target-6 (ESAT6) antigens are secreted and the detection of such proteins can be used for the early and specific diagnosis of tuberculosis [85]. Therefore, Tang et al. [84] raised aptamers against CFP10 and ESAT6 antigenic targets using SELEX. The selected screened aptamers (CE24 and CE15) were used in an enzyme-linked oligonucleotide assay (ELONA) to detect the proteins CFP10 and ESAT6 in serum samples of patients with active pulmonary tuberculosis, extra-pulmonary tuberculosis and healthy donors. The results demonstrated a specificity and sensitivity of 94.1% and 100% (using CE24-based ELONA) and, 94.1% and 89.6% (using CE15-based ELONA), respectively [84].

Recently, aptamers have also been conjugated with MNPs to improve diagnosis. For example, Wang et al. [86] developed POC diagnostic systems to diagnose sepsis and perform blood disinfection using aptamers. The commercial aptamers able to recognise bacterial species were conjugated with iron oxide MNPs functionalised with chlorin e6 (Fe_3_O_4_-Ce_6_-Apt). This nano-system allowed successful diagnosis of sepsis in mouse models caused only by *S. aureus* or by multiple bacterial species, namely *S. aureus* and *E. coli*, with a detection sensitivity comparable with serological techniques and a shorter turnaround time. Moreover, a total extracorporeal disinfection of blood was achieved due to the strong photodynamic effect of the Fe_3_O_4_-Ce_6_-Apt system. Another example of Fe_3_O_4_ MNPs functionalised with aptamers was reported by Hao et al. [87] for the specific capture of enteropathogenic *S. typhimurium*. Commercially available specialised aptamer complexes containing a sequence specific to capture *S. typhimurium* and a primer region used to assist in detection were used. These primer sequences play a role in rolling circle amplification (RCA) to produce long ssDNA with hundreds of tandem-repeat sequences [88]. Therefore, while the bacteria are captured, simultaneously several signal probes are assembled on the RCA products for enhanced amplification of the recognition event. This unique method was reported to have a high selectivity for *S. typhimurium*, with an excellent LOD equal to 10 CFU/mL.

In addition to the abovementioned studies, there are currently many other examples of aptamers used to identify various pathogens and their biomarkers [89,90,91]. Table 2 and Table 3 summarise other examples of aptamers used to identify human bacterial pathogens and viral human pathogens, respectively, for the diagnosis of infections.

Despite aptamers’ numerous advantages and recognised potential, the translation of aptamer-based products to the clinics or other markets has been slow [118,119]. Reasons for this include the high financial investment made in the research and production of antibodies, and the general unfamiliarity regarding aptamers and their interesting performance [120]. In addition, although aptamers may be cheaper to produce, in general, the affinity properties of antibodies in comparison to aptamers remain superior. Furthermore, SELEX can be a very time-consuming process. However, their advantages together with an increasing awareness about them could lead, in the near future, to a wider use of aptamers in the increasingly relevant POC diagnostics and therapeutics field.

#### 3.4.2. DNAzymes

DNAzymes, also called deoxyribozymes, are synthetic ssDNA oligonucleotides that display catalytic activities [121]. Inspired by the existence of naturally occurring ribozymes (RNAzymes), Breaker and Joyce identified, in 1994, the first DNA-like enzymes by SELEX [122]. DNAzymes were initially produced for the detection of lead contaminations [123]. More recently, they have been generated to identify cancer cells [124], pathogenic bacteria [125] and other biomarkers. For example, Zheng et al. [125] created a sensing platform using MNPs functionalised with a fluorescently-responsive DNAzyme for the detection of pathogenic *E. coli*. The *E. coli*-specific DNAzyme was synthesised by template-mediated ligation and further modified with MNPs and acetylcholinesterase (AChE) to form a complex. In the presence of bacterial lysate, the DNAzyme domain binds to target molecules from the bacterial content, triggering a cleavage event which releases AChE. The free AChE subsequently plays a role in enhancing the fluorescence signal of the detection system. DNAzymes were found to bind to the target with high specificity and sensitivity, exhibiting a LOD of 60 CFU/mL with a linear range from 10^2^ to 10^7^ CFU/mL. 

**Figure 5:**
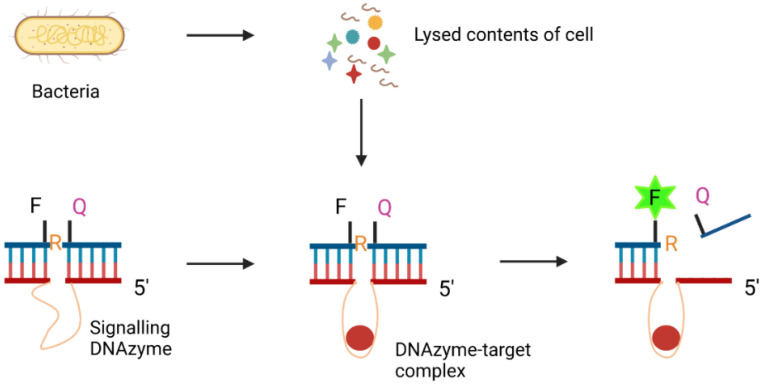
DNAzyme biosensor proposed by Kang et al. [126]. The target(s) from lysed bacteria bind(s) to the DNAzyme sequence (orange), which undergoes a change in conformational triggering the activation of the DNAzyme. The activated DNAzyme cleaves the fluorogenic substrate at the ribonucleotide connection (R), this releases the fluorophore (F) and quencher (Q) to produce a high-fluorescence signal. Created with BioRender.com.

A highly innovative system for the detection of bacteria in blood was also developed by Kang et al. [126]. SELEX was used to identify DNAzymes with specificity to the *E. coli* lysates. The construction of the DNAzyme is shown in Figure 5. The DNAzyme domain was enzymatically ligated with a DNA–RNA chimeric substrate. This substrate contained a ribonucleotide cleavage site flanked by a fluorophore and a quencher. In the presence of the target *E. coli* lysate, the DNAzyme binds the target molecule, changes its conformation and cleaves the fluorophore from its quencher, generating a high fluorescence detectable signal. During detection, DNAzyme sensors were mixed with blood constituents and then encapsulated in hundreds of millions of picolitre droplets. These were analysed by a 3D high-throughput particle counter to detect fluorescent particles. Using this method, *E. coli* was detected in a range of very low concentrations from a single cell up to 10^4^ cells per mL within a span of 1.5 to 4 h. Afterwards, this biosensor was adapted for detection of *Klebsiella pneumoniae* in a fluorescent paper sensor [127].

DNAzymes were also successfully employed in a colorimetric paper sensor for the sensitive detection of another human pathogen, *Helicobacter pylori*, from human stool samples achieving a LOD of 10^4^ CFU/mL [128]. The RNA-cleaving properties of the DNAzyme were activated by a protein biomarker of *H. pylori* and the DNAzyme was identified by an in vitro selection process similar to SELEX. The method required minimal sample processing and was completed in a few minutes.

In relation to virus detection, Kim et al. [129] devised a fast and simple colorimetric assay to detect the human immunodeficiency virus (HIV) from human serum using a previously established functional DNAzyme motif generated by conventional PCR. The key aspect is the design of the primers that target the HIV-1 *gag* gene and, which during PCR amplification, insert a functional DNAzyme sequence in the PCR product. After amplification, hemin is added to assist in the formation of a G-quadruplex structure within the DNAzyme sensor to catalyse the oxidation of 2,2′-azino-bis(3-ethylbenzothiazoline-6-sulfonate) (ABTS). The oxidation of ABTS yields a change from colourless to blueish green which can be visualised and quantified. Recently, Anantharaj et al. [130]. designed a biosensor for the detection of SARS-CoV-2 RNA using the same working principle. This DNAzyme sensor is highly sensitive because it selectively targets the N gene of SARS-CoV-2, which is not present in the genomes of other viruses. The LOD of the sensor was 10^3^ copies of viral RNA. Other recent advances of G-quadruplex DNAzyme based biosensors were revised by Xi et al. [131].

Similar to aptamers, DNAzymes exhibit remarkable selectivity to their targets and therefore continue to find use as recognition molecules for a wide range of applications.

#### 3.4.3. Peptide Nucleic Acids (PNAs)

PNAs are artificial molecules composed of a polypeptide backbone with nucleic acid bases attached as side chains. They are notable nucleic acid analogues due to their unique physicochemical and biochemical attributes, stability and striking hybridisation attributes [74]. Due to these characteristics, PNAs have wide application in molecular diagnosis [132].

A paper-based colorimetric multiplex sensor using a Pyrrolidinyl PNA (acpcPNA) probe for the detection of Middle East Respiratory Syndrome coronavirus (MERS-CoV), human papillomavirus (HPV) and *M. tuberculosis* was successfully developed by Teengam et al. [133]. The probe was based on a previously described motif [134], wherein a PNA conjugated with modified AgNPs induces aggregation of the AgNPs in the absence of complementary target DNA. When the target DNA is present, a DNA–acpcPNA duplex is formed, originating the dispersion of the AgNPs and a concomitant detectable colour change.

PNA Fluorescence In Situ Hybridisation (PNA-FISH) has become useful for the specific, reliable and rapid detection of human pathogens. Machado et al. [135] developed a novel PNA-FISH method with specificity for *Lactobacillus* and *Gardnerella vaginalis*. Specificity and sensitivity of the PNA probes were 98% and 100% for *Lactobacillus* and *G. vaginalis,* respectively. Furthermore, the probes were evaluated in samples mimicking the vaginal microflora of patients with a bacterial vaginosis infection, to demonstrate their applicability for diagnosis. PNA-FISH was also recently used by Rocha et al. [136] in the detection of *L. monocytogenes* using a previously developed PNA probe (LmPNA1253) selected by Almeida et al. [137] coupled with a new blocker probe. The method was able to detect *L. monocytogenes* with an LOD of 0.5 CFU/mL in certain food samples.

PNA probes have also been used in the detection of clinical viruses, such as the hepatitis C virus (HCV), which causes chronic liver disease. Ahour et al. [138] developed an electrochemical sensor for HCV detection based on a 20-mer PNA probe that targets a highly conserved consensus sequence present in core/E1 domain from HCV genome. This sequence was cloned in a recombinant plasmid that was used to test the electrode. The PNA was able to hybridise to this sequence without being necessary to denature the plasmid. This represents an advantage because the DNA in nature is in a double-stranded form. Therefore, this method can be used for identification of all HCV genotypes through direct detection. For detection, a gold (Au) electrode modified with cysteine and conjugated with the PNA probe was used. The hybridisation detection was performed by monitoring the difference between the voltametric response of methylene blue (which serves as an electroactive indicator) accumulated on the PNA–modified Au electrode before and after the hybridisation event.

It can be concluded that PNAs hybridise more efficiently with complementary DNA and RNA because they are neutral and their interactions lack charge repulsion which is present in other nucleic acid-based probes. Therefore, they are highly useful when used in conjunction with technologies such as FISH to create simple, rapid and highly accurate microbial detection sensors [139]. In addition, sensors employing PNAs have also been suggested to have great potential in the detection and diagnosis of COVID-19, and can thus aid in curtailing its spread [140].

### 3.5. CRISPR-Cas

CRISPR are a family of DNA sequences originating in bacteriophages that have previously infected prokaryotes and subsequently become incorporated into their genomes as a defence mechanism to recognise foreign nucleic acid sequences and eliminate them by using the endonuclease activity associated with an enzyme called Cas. Cas evolved in prokaryotes for defence against invading viruses by cleaving their nucleic acid like a pair of scissors [141,142]. CRISPR RNA (crRNA) guides Cas to recognise and cleave target nucleic acids; thus, crRNA can be programmed towards any specific DNA or RNA of interest such as pathogenic genetic material, for instance, by hybridising to a complementary sequence [143]. In this manner, CRISPR-Cas has been repurposed as a gene editing tool, in disease treatment and diagnosis [144]. For diagnostic applications, CRISPR-Cas systems have been used to sense nucleic acid-based pathogenic biomarkers with single-base resolution.

An example of such a system is CRISPR-Cas9, which leverages its sequence-specific nuclease activity to distinguish between viral lineages. To illustrate, a recent Zika virus outbreak prompted Pardee et al. [145] to develop a widely regarded workflow for a portable, low-cost colorimetric sensor which couples isothermal RNA amplification and toehold switches on a paper-based platform. The sensor was further coupled with a CRISPR-Cas9 module that has the ability to distinguish between strains of a virus with single-base resolution. Briefly, in the presence of the target RNA, a Cas9-mediated cleavage is triggered, resulting in a truncated RNA product that is unable to activate the sensor toehold switch and does not produce a colour change in the test paper. While in the presence of non-target viral RNA, the full-length RNA product comprising the sequence to trigger the sensor is generated, thus activating the sensor and producing a colour change on test paper. Detection of Zika virus from monkey plasma infected with the virus was achieved in the low femtomolar range. Additionally, Ai et al. [146] also developed a rapid assay using CRISPR for detection of *M. tuberculosis.* The method combined an amplification step of the target bacterial sequence by recombinase polymerase amplification (RPA) followed by a Cas12a detection step. After the amplification step, the presence of target RNA activates the Cas12a cleavage system, which in turn triggers a colour change in an ssDNA reporter that is present. The detection system is extremely sensitive with almost single-copy sensitivity. More recently, Kellner et al. [147] established a platform that also combines RPA with CRISPR-Cas to detect target RNA or DNA sequences. This platform was named SHERLOCK, which stands for specific high-sensitivity enzymatic reporter unlocking. In addition to being portable and extremely sensitive in the detection of DNA or RNA from real clinical samples, this platform is able to detect multiple targets through the use of a multiplex fluorescence-based detection system.

**Figure 6:**
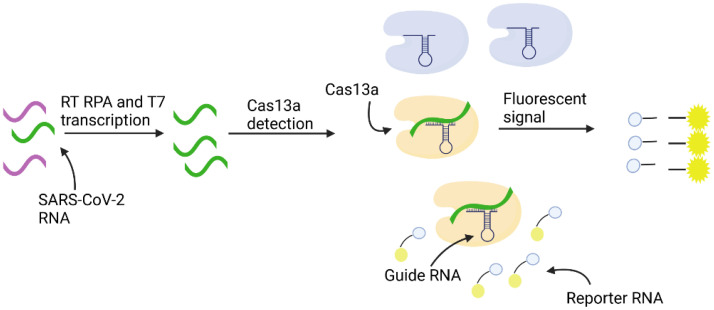
Schematic illustration of the Clustered Regularly Interspaced Short Palindromic Repeats/Cas13a (CRISPR/Cas13a) system for detection of SARS-CoV-2 ribonucleic acid (RNA) proposed by Hou et al. [148]. Reverse-Transcription Recombinase Polymerase Amplification (RT-RPA) followed by a T7 transcription is used to amplify the SARS-CoV-2 RNA. In the next step, the nuclease activity of Cas13a is activated when the guide RNA binds specifically to the open reading frame 1ab (*orf1ab*) gene and triggers the cleavage of the RNA reporter. The cleaved RNA reporter produces a fluorescent signal for detection of SARS-CoV-2. Created with BioRender.com.

For the detection of SARS-CoV-2, Hou et al. [148] proposed an alternative to the standard reverse transcription quantitative polymerase chain reaction (RT-qPCR) detection by means of a rapid assay based on polymerase-mediated amplification and CRISPR/Cas13a. Figure 6 illustrates the steps of the assay. This isothermal method is highly advantageous because it does not require expensive and bulky thermocycler equipment and only takes 40 min. To test the novel assay, 52 RNA samples from patients with COVID-19 were subjected to mNGS and three potential target sequences were identified. Subsequently, CRISPR gRNAs and RPA primers were designed and screened. A primer set that targeted open reading frame 1ab (*orf1ab*) displayed the best specificity and sensitivity and was used to develop the CRISPR assay, which was based on T7 transcription and a Cas13 detection step. To evaluate the specificity of the CRISPR assay, target viral DNA was substituted with human DNA and a panel of bacterial and viral pathogens. None of these test samples caused a false positive reaction. Further, the CRISPR assay demonstrated 100% sensitivity because it was able to detect all 52 cases of COVID-19. In the future, the role of CRISPR-associated nucleases can be expanded for direct diagnostic testing of nucleic acids due to their exceptional single molecule sensitivity.

### 3.6. Bacteriophages

Bacteriophages (also termed phages) are a type of viruses that infect and replicate within their target bacteria. Due to their high specificity, conferred by receptor binding proteins (RBPs) on the bacteriophage surfaces with which they target bacteria, they have potential for application in diagnostic tools and treatments against bacterial infection [149,150,151,152]. For instance, Liana et al. [153] functionalised MNPs with a high density of T4 bacteriophages and subsequently used them to capture *E. coli.* T4 bacteriophages infect *E. coli* and the one used in the reported study had specificity to *E. coli* type B (ATCC 11303) by means of their tail fibres. In addition to the density of bacteriophage loaded on the MNPs, the authors reported the important effects of tryptone presence in the medium and the incubation temperature used to grow *E. coli* in the capture capabilities of the MNP probes. The T4 bacteriophages were found to bind irreversibly to *E. coli* at 37 °C in tryptone-containing media (rich in tryptophane) during an incubation time of just 10 min.

In a different study, tosyl-activated MBs were functionalised with a bacteriophage PAP1, which is highly specific to *P. aeruginosa*, to establish a bacteriophage-affinity strategy for its detection [151]. The bacteriophage tail fibres and baseplate identified and captured *P. aeruginosa* onto the MBs. Subsequently, the bacteriophage replication cycle proceeded for about 100 min after which the progenies lysed the bacteria causing the release of intracellular ATP. A firefly luciferase-ATP bioluminescence system was used to quantify the captured bacteria. The LOD was determined to be 2 × 10^2^ CFU/mL and the process of capture and detection was completed within 2 h. To estimate the suitability of this strategy for POC diagnosis, glucose, human urine and rat plasma samples were spiked with *P. aeruginosa* at various concentrations and bacteria recovery tests with the functionalised MBs were performed. Recovery rates ranged from 77.4% to 96.9%, demonstrating good reliability for the detection of bacteria in complex samples [151]. In an example of multiplex detection [150], two phage RBPs, *gp*18 and *gp*109, with potential specificity to the genera *Enterococcus* or *Staphylococcus*, respectively, were identified in silico. The RBPs were fused with fluorescent proteins and used in spectrofluorometric assays with the target bacteria for their multiplex detection by fluorescence. Additionally, *gp18* also showed high sensitivity to *E. faecium* and *E. faecalis* by binding to 80% and 100% of tested strains, respectively.

The exceptional specificity displayed by bacteriophages for their target render them prime candidates for diagnostic platforms. However, more bacteriophages that target other dangerous pathogens need to be further discovered or engineered to build promising diagnostic systems that can also function as therapeutics.

### 3.7. Molecularly Imprinted Polymers (MIPs)

Artificial material-based biorecognition elements rely on the specific morphology or shape of the target for selective capture [11,154]. MIPs include cell imprinted polymers (CIPs), which are the most common examples of MIPs in the context of biorecognition. Some processes used to produce MIPs include micro-contact stamping, bacteria-mediated lithography and colloid imprints. For example, Khan et al. [155] fabricated a MIP by imprinting the bacterial flagella of *P. mirabilis* onto electropolymerised phenol. The flagellar protein imprint sites have rebinding ability in the presence of a sample containing *P. mirabilis*. The MIP was evaluated for biorecognition in an electrochemical biosensor to detect *P. mirabilis* during a rebinding event. The sensor was found to be highly sensitive with an LOD of 0.7 ng/mL.

There are many more examples on the use of CIP in diagnosis. For example, Golabi et al. [156] reported an electrochemical biosensor employing CIPs that specifically recognise *S. epidermidis.* The CIPs’ fabrication is based on the polymerisation of 3-aminophenylboronic acid. The features of the imprinted surface include complementary cavities at the polymer surface, presenting structural specificity in terms of shape and size, but also chemical specificity via diol molecules which are present on the cell walls of *S. epidermidis*. The sensor response was reported as being proportional to log 10^3^–10^7^ CFU/mL of *S. epidermidis* and was highly specific for the target strain when compared to non-target species such as *E. coli, Deinococcus proteolyticus* and *S. pneumoniae*. Similarly, a polydopamine-based CIP was imprinted with template *E. coli* O157:H7 for capturing bacteria together with a polyclonal antibody [157]. By electro-chemiluminescent detection, a very low LOD of 8 CFU/mL was measured. There are other successful demonstrations of bacterial biorecognition by employing CIPs reporting high specificity and sensitivity [158,159].

CIPs are also widely applied in the detection of virus in diagnostics [160]. Furthermore, Cai et al. [161] developed an MIP-based sensor that exhibited exceptional specificity for E7 protein derived from the HPV [162]. The MIP was built by imprinting the tips of nanotube arrays with a polyphenol nanocoating. The coating was non-conducting and detection was performed by electrochemical impedance spectroscopy. The high specificity of the sensor was confirmed when another similar protein of HPV called E6 was not recognised by the E7 MIPs. The E7 protein was recognised with sub picogram per litre sensitivity, surpassing that achieved by conventional MIPs and comparable with nanosensors based on biomolecular recognition with ligands. A similarly constructed MIP system was developed by Ma et al. [163] for specific recognition of the HIV-p24 capsid protein of the HIV virus.

Detection by MIPs is label-free and can be even more affordable than ligand-based biorecognition platforms. However, during a binding event between template and molecules, conversion of the resulting signal by detection systems needs to be improved, in addition to elimination of noise in the signal.

### 3.8. Antibiotics

Although most antibiotics lack high selectivity due to their broad-spectrum activity, in combination with certain upgrades that provide or improve selectivity, they have proven to be effective for biorecognition of pathogenic targets, as illustrated by the examples presented in this section [164,165]. Antibiotics are widely available, highly stable and exhibit strong binding capabilities to bacteria, making them excellent for combinatorial strategies in biorecognition or in pre-enrichment steps in sensing platforms [164]. However, this approach can only be considered for bacterial species that have not developed resistance to the antibiotic under consideration.

#### 3.8.1. Vancomycin

Vancomycin (Van) is a well-known broad-spectrum lycopeptide antibiotic that interacts with a range of Gram-positive bacteria. Lycopeptide antibiotics are known to bind to the D-alanyl-D-alanine (D-Ala-D-Ala) dipeptide groups present in the cell wall of bacterial strains through hydrogen bonds [166]. Therefore, Van is popularly used in the functionalisation of MBs as bacterial capture probes and in combination with secondary recognition molecules to improve specificity [164]. For example, Yang et al. [167] designed multivalent “brush-like magnetic nanoprobes” and demonstrated their potential for the efficient enrichment of pathogens. To construct the brush-like magnetic nanoprobes, commercial amino-MBs were modified with poly-L-lysine (PLL), followed by the connection of polyethylene glycol (PEG) to the amine sites of PLL. Van was previously linked to the carboxyl molecule of PEG. By using these nanoprobes, an enrichment efficiency greater than 94% was achieved, and also an excellent recovery of *L. monocytogenes* was obtained within 20 min, at a bacterial concentration of 10^2^ CFU/mL. Moreover, Meng et al. [168] synthesised similar PEGylated MNPs functionalised with Van (Van-PMs), also to capture and enrich the virulent foodborne pathogen *L. monocytogenes* from spiked lettuce samples prior to detection by PCR. The Van-PMs displayed a high capture efficiency of around 83% and 90% with LOD of 30 CFU/g and 30 CFU/mL in lettuce samples and in PBS, respectively. All steps, including enrichment and PCR, were accomplished in 4 h.

On another study, Yang et al. [164] synthesised MBs with a size of 100 nm functionalised with Van to capture *S. aureus* prior to chemiluminescent detection. To improve the specificity of these Van-MBs to *S. aureus*, rabbit Ig G (IgG) tagged with ALP was used as a second recognition molecule, because the Fc region of rabbit IgG binds to protein A in *S. aureus* surface. The resulting sandwich complex of Van-MBs-*S. aureus*-IgG significantly improved the specificity due to the recognition of *S. aureus* at two distinct sites. In addition, it facilitated ultrasensitive chemiluminescent detection of *S. aureus* in a linear range of 1.2–12 × 10^6^ CFU/mL and with a very low LOD (3.3 CFU/mL). The entire process was completed in 75 min.

More recently, Wang et al. [169], developed an efficient SERS biosensor that combined Van-modified Fe_3_O_4_ AgNPs and gold and silver (Au-Ag) NPs for enrichment and sensitive and specific detection of bacteria. The high-performance Van-Fe_3_O_4_@Ag MNPs served as effective capture probes for Gram-positive bacteria such as *E. coli*, *S. aureus* and methicillin-resistant *Staphylococcus aureus* (MRSA), achieving a LOD of 5 × 10^2^ cells/mL. Then, the plasmonic Au-Ag NPs were used as secondary NPs to increase the detection sensitivity.

#### 3.8.2. Amoxicillin

Some bacteria possess so-called penicillin binding proteins (PBPs) which generate β-lactamase enzymes to resist β-lactam producing antibiotics such as amoxicillin and penicillin by breaking the lactam amide bond. Due to this interaction, the β-lactam in amoxicillin has selective affinity to PBPs present on the bacterial cell membrane [170]. Based on this chemical interaction, Hasan et al. [171] synthesised an effective enrichment probe against *S. aureus* and *E. coli* by modifying MNPs with 3-aminopropyltriethoxysilane and amoxicillin to target PBPs. The modified MNPs successfully captured bacteria forming aggregates that are then separated using an external magnet. MALDI-TOF was used to confirm the interaction between the amoxicillin and bacterial PBPs. The LOD for *S. aureus* and *E. coli* was in the range of 10^3^–10^4^ CFU/mL using MALDI-TOF after 5 min incubation with amoxicillin functionalised MNPs.

#### 3.8.3. Ampicillin

Ampicillin has all the stated advantages of antibiotics for use in biorecognition. It is widely available, bringing down costs, and has broad-spectrum high-affinity binding properties for bacteria. These strong affinity properties of ampicillin and the specificity of antibodies were combined to design a LFA for detection of *Salmonella enteritidis* [165]. Ampicillin coated on MNPs facilitated the initial enrichment of *S. enteritidis* from spiked food samples by capture and magnetic separation. Subsequently, the enriched sample was applied to the LFA. The anti-*S. enteritidis* mAB with high specificity towards *S. enteritidis* dispensed on the LFA functioned as a specific enrichment agent, forming a sandwich complex with the ampicillin-MNPs. The colour change was detected by the naked eye at an LOD of 10^2^–10^3^ CFU/mL.

#### 3.8.4. Neomycin

Neomycin is an aminoglycoside broad spectrum oligosaccharide antibiotic that targets the 30S ribosomal subunit and interferes with decoding and translocation, which, consequently, inhibits protein synthesis in bacteria [172]. Zhang et al. [173] developed lipid conjugated neomycin-based probes that selectively identify and label antibiotic-resistant bacteria simultaneously without disrupting host immune cells. In addition, this probe has theranostic abilities, such as the presence of neomycin, which inactivates the bacteria in addition to detection. This is useful because the probe can have therapeutic and diagnostic applications [10]. The lipidated probes exhibited highly specific and strong fluorescent signals against MRSA and an increased inhibition effect compared to probes with only neomycin. This increased specificity and decreased bacterial resistance is related to lipid chains being able to increase the membrane permeability to the antibiotic. Therefore, this strategy has the potential to detect bacterial infections with high selectivity and sensitivity, and enhance the antibacterial effect of antibiotics without harm to host cells.

Clearly, a number of antibiotics have been tested for their potential as capture probes and, in combination with selective ligands, can be very effective for bacterial identification.

### 3.9. Chemical Compounds

In addition to the classes of biorecognition elements exhaustively discussed above, various molecules not covered by these classes also exhibit affinity and selectivity for pathogens and can be exploited for use in sensing platforms. In one such example, Pang et al. [174] designed a platform partly composed of maltohexaose-decorated cholesterol that is able to target bacteria both in vitro and in vivo through a bacteria-specific maltodextrin transporter pathway. They investigated the theranostic capabilities of this smart nanoliposome-based platform (MLP18) for targeting of multi-drug resistant strains of MRSA and β-lactamase *E. coli* in different mouse models and subsequent delivery of purpurin 18 (P18), a sonosensitiser for sonodynamic therapy. After injection in a mouse-model, the MLP18 selectively directs P18 to the bacterial infection site. Fluorescence imaging showed that the MLP18 produced a specific fluorescence signal at the site of infection that remained for 24 h post-injection. At the site of sterile inflammation only a negligible fluorescence signal was detected, demonstrating the bacterial-targeting abilities of MLP18.

Moreover, Fu et al. [175] investigated the considerable potential of chemical ligand discovery from natural sources by the coupling of affinity MS and metabolomic approaches. They identified two ligands, 18β-glycyrrhetinic acid and licochalcone A derived from liquorice root used in traditional Chinese medicine which were able to bind and disrupt the nucleoprotein of EBV and Marburg virus. Ligand binding assays and various biophysical analyses were used to evaluate the interactions of the ligands with the bacterial nucleoproteins.

During infection, pathogens interact with host tissue and some of these interactions are specific, such as when pathogens bind to specific host cell membrane proteins. For example, human extracellular matrix proteins, such as collagen and fibronectin, are known receptors for outer membrane protein adhesins present on various microbial pathogens during the initial stages of infection [176]. These interactions can be exploited by coating collagen or fibronectin on MBs, for example, and using them to capture and detect pathogens using sensing platforms.

## 4. Future Perspectives

A paradigm shift is taking place in the way infectious diseases, predominantly caused by pathogenic bacteria and viruses, are being diagnosed. However, even though highly technological methods that can highly sensitively identify pathogens from samples emerged a few decades ago, this has not been translated into a faster, more efficient and accurate diagnosis for most people suffering with various infections due to their inaccessibility. As discussed, it is evident that diagnostics in the form of simple POC tests, biosensors and kits are the future and can greatly improve outcomes for patients due to a timely diagnosis. Development of workflows to detect the myriad pathogens is underway and is dominated by various sensing platforms. In this review, the latest biorecognition elements in workflows and detection systems are categorised and a number of recent application examples for each are elucidated. The biorecognition elements that hold the greatest potential for incorporation into POC diagnostics are the nucleic acid derivatives, if they can equal antibodies in performance parameters. The incorporation of such elements can drive down the cost of biosensors. Antibody-based sensors will continue to be popular but their market share may shrink. Alternatively, CRISPR technology has an emerging potential for diagnostics among all the biorecognition elements herein discussed due to their impressive sensitivity. However, extensive research is needed for each infectious disease to choose the right combination of Cas enzyme and to design the best system.

Nevertheless, there are some bottlenecks in the translation of sensing platforms comprising these biorecognition elements from bench-top to clinical. A vast array of development in the field is available, although not well organised. There is no standard method to determine which platforms have the potential to achieve commercial development and need more funding, for instance. Furthermore, biorecognition elements need to be subject to some kind of standardisation procedures, in addition to parameters related to their targets, such as LOD values. Collaboration between academia, clinical practitioners and industries that can invest in the further development of POC sensing platforms is critical, followed by consideration of regulatory clearance and introduction to commercial markets.

## 5. Conclusions

In conclusion, based on the evidence presented in this review, antibodies currently remain the biorecognition element of choice, but alternatives, especially in the form of nucleic acid derivatives, are being sought. 

Antibodies are still dominantly used in commercial immunoassays and immunosensors, and are at various stages of research. This is due to their ability to provide a combination of strong target affinity and exceptional selectivity. Furthermore, due to their extensive use over many decades, the field of antibody selection is well developed with a vast number of molecules available and well characterised for selection based on the envisaged application. However, alternatives are increasingly being sought due to their disadvantages, including a cumbersome development and selection process that is expensive, in addition to their inherent instability, which can make them difficult to incorporate in sensor kits. The advantages and disadvantages of using enzymes as biorecognition elements are similar to those of antibodies. Due to their natural origin, they display superior affinity properties to targets, but they also suffer from instability issues, and are difficult to isolate and process in a laboratory environment. Possibly due to this issue, the implementation of enzymes as biorecognition elements for diagnostic applications is still limited and needs further development.

As mentioned, the main alternatives to antibodies being studied are derivatives of nucleic acids such as aptamers, PNAs, DNAzymes and antibody-derived fragments. The main advantage of those molecules is that they can be developed much more cheaply than antibodies. In some instances, they have demonstrated target affinity comparable with their antibody counterparts while exhibiting excellent stability and reproducibility, which is a vital requirement for POC diagnostics. They are also versatile enough to be combined with most POC sensor detection platforms, including electrochemical, optical, colorimetric and LFA. Their main disadvantage is that they require a selection process that is time-consuming and sometimes difficult to perform. However, as illustrated in this review, there are numerous excellent examples of nucleic acid derivative-based biorecognition elements in sensing platforms, which will certainly translate to their extensive application in POC sensors in the near future. 

Classes of compounds and molecules such as antibiotics and peptides, which were traditionally used for anti-infective and therapeutic purposes, have seen their broad-spectrum activity against pathogens re-purposed for pathogen identification in diagnostics. Due to their wide range, they are usually used for pre-enrichment or combined with other biorecognition elements to enhance the sensitivity of the sensor. A huge number of AMPs are under scrutiny; however, their emerging disadvantage is the lack of selectivity.

Bacteriophages present excellent selectivity due to their innate targeting function, which is similar to that of antibodies. Development of bacteriophage-based sensing platforms is in a nascent stage compared to the other biorecognition elements, but their high target specificity makes them promising candidates for biorecognition. 

MIPs have been included in this review due to their importance and demonstrated capabilities in pathogen-sensing platforms, although ligand molecules are not usually used for biorecognition in these cases. Their advantages include lower production cost compared to antibodies, high batch-to-batch reproducibility, and chemical and mechanical robustness, and do not involve animals’ sacrifice. MIPs can be extremely versatile because almost any target can be imprinted for their detection and have great potential in sensing platforms. 

CRISPR-Cas technology, although still emerging in the diagnostic landscape, has already shown immense potential with platforms such as SHERLOCK-enabled multiplexed and ultra-sensitive detection of DNA or RNA from clinical samples. The main advantage of CRISPR is that its single-base resolution selectivity is unmatched by any other biorecognition element. The nature of the technology enables it to be leveraged in POC diagnostic sensing platforms. Finally, chemical compounds such as metabolites, polysaccharides and several other chemicals are useful for targeting specific pathogenic targets for which they have a known affinity interaction. In conclusion, depending on the application, one or a combination of more suitable biorecognition element(s) investigated in this review can provide high performance biorecognition capabilities in POC diagnostics.

## Figures and Tables

**Table 1:** Commercially available clinical biosensors for identification of human pathogens.

Pathogen Class	Biorecognition Element	Company	Product	Type of Test	Refs.
*Clostridium difficile*	Antibodies specific to *C. difficile* antigen glutamate dehydrogenase	Corisbio	*Clostridium* K-SeT	Immunochromatographic assay	[14]
	N/A	Thermo Scientific	Oxoid™ *Clostridium difficile* Test Kit	Rapid latex agglutination	[15]
*Escherichia coli* serogroup O157	N/A	Meridian Bioscience, USA	ImmunoCard STAT! *E. coli* O157 Plus	Lateral flow immunoassay	[16]
	Antibodies specific to O157 serogroup antigen	Thermo Scientific	*Escherichia coli* O157 Latex Test	Colorimetric latex agglutination	[17]
*Haemophilus influenzae b*	Antibodies specific to *H. influenzae* type b antigen	Thermo Scientific	Wellcogen™ *Haemophilus influenzae* b	Rapid Latex Agglutination	[18]
*Helicobacter pylori*	Antibodies specific to *H. pylori*	Quidel, USA	QuickVue *H. pylori* Test	Solid phase immunoassay	[19]
	Anti-*H. pylori* lgG antibody	Beckman Coulter, USA	Icon HP	Immunoassay	[20]
	Antibodies specific to *H. pylori* antigens	Corisbio	Pylori-Strip/Pylori K-SeT	Immunochromatographic assay	[21,22]
*Moraxella catarrhalis*	Indoxyl butyrate detects the enzyme butyrate esterase of *M. catarrhalis*	Thermo Scientific	Remel™ *Catarrhalis* Test Disc	Colorimetric chemical identification	[23]
*Staphylococcus aureus*	A protein detects clumping factor and protein A of *S. aureus*	Thermo Scientific	BactiStaph™ Latex Agglutination Test Kit	Agglutination of protein-coated latex particles	[24]
*Streptococcus pneumoniae*	Specific rabbit antibody	Thermo Scientific	DrySpot™ Pneumo Latex Agglutination Test	Agglutination of antibody-coated latex particles	[25]
	N/A	Abbott	BinaxNOW™ *Streptococcus pneumoniae* Antigen Card	Lateral flow immunoassay	[26]
Epstein-Barr virus	Heterophile antibody detects bovine red cell mononucleosis antigen	Thermo Scientific	Infectious Mononucleosis Test using Latex Agglutination	Latex Agglutination	[27]
Influenza A and B virus	Antibodies specific to Influenza type A and type B antigens	Quidel, USA	QuickVue Influenza A + B test	Immunoassay	[28]
	Antibody detects nucleoprotein antigens	Abbott	Alere BinaxNOW^®^ Influenza A & B Card	Immunochromatographic assay	[29]
Severe acute respiratory syndrome coronavirus 2 (SARS-CoV-2)	N/A	Lucira Health	COVID-19 All-In-One Test Kit (FDA approved)	Amplification of viral genetic material	[30]
	Antibody	Abbott	BinaxNOW COVID-19 Ag Card Home Test (FDA Emergency Use Authorization)	Rapid antigen	[31]
	Antibody	Ellume Health	Ellume COVID-19 Home Test (FDA Emergency Use Authorization)	Rapid antigen	[32]
*Plasmodium falciparum*	Antibodies detect histidine-rich protein II antigen specific to *P. falciparum* and a pan-malarial antigen	Abbott	BinaxNOW^®^ Malaria	Immunochromatographic assay	[33]

N/A—Information Not Available.

**Table 2:** Summary of recent aptamer-based methods for the identification of bacterial pathogens.

Bacteria	Target (Analyte)	Detection System	Refs.
*Escherichia coli* O157:H7 and *Salmonella typhimurium*	Whole bacteria	Aptamer-modified fluorescent magnetic multifunctional nanoprobes	[92]
*Staphylococcus aureus* and *Escherichia coli*	Whole bacteria	Multiplex aptamer-based hydrogel barcodes	[93,94]
*Escherichia coli* O157:H7	Whole bacteria	Electrochemical biosensor with amino-functionalised metal–organic frame integrated with aptamers	[95]
*Staphylococcus aureus*	Whole bacteria	Electrochemical detection by dual-aptamer-based sandwich with silver nanoparticles	[96]
*Staphylococcus aureus*	Enterotoxin A protein	Graphene oxide-based fluorescent bioassay	[97]
*Staphylococcus aureus*	Whole bacteria	Surface-enhanced Raman spectroscopy (SERS) biosensor	[98]
*Campylobacter jenuni*	Whole bacteria	N/A	[77]
*Streptococcus pyogenes*	M11 M-type serotype whole bacteria	N/A	[99]
*Mycobacterium tuberculosis*	MPT64 secreted protein	Enzyme linked oligonucleotide assay	[100]
Methicillin-resistant *Staphylococcus aureus* (MRSA)	Penicillin binding protein 2a (PBP2a)	Fluorometric assay	[101]
*Pseudomonas aeruginosa*	Whole bacteria	Fluorometric assay	[102]

N/A—Not Applicable.

**Table 3:** Summary of recent aptamer-based methods for the identification of viral pathogens.

Virus	Target (Analyte)	Detection System	Refs.
Avian influenza strain H5Nx	Whole virus	Sandwich-type surface plasmon resonance (SPR)-based biosensor assay	[103]
Influenza A strain H3N2	Globular region of hemagglutinin	Aptamer-functionalised magnetic microparticle-based colorimetric method	[104,105]
Zika	Zika NS1 Protein	Aptamer-Based enzyme-linked immunosorbent assay (ELISA)	[106]
Norovirus	Murine norovirus and capsids of a human norovirus strain GII.3	Electrochemical sensor	[107]
H1N1	Inactivated H1N1 virus particles	Electrochemical impedance sensor	[108]
Human immuno-deficiency virus Type 1 (HIV-1)	Glycoprotein-120 (gp-120)	Liquid crystal optical sensor	[109,110]
Human papillomavirus (HPV)	L1-major capsid protein of HPV	Electrochemical impedance sensor	[111,112]
SARS-CoV-2	Receptor-binding domain (RBD) of the spike glycoprotein	N/A	[113]
SARS-CoV-2	Nucleocapsid protein	ELISA and a gold nanoparticle immunochromatographic strip	[114]
SARS-CoV-2	Nucleocapsid protein	N/A	[115]
SARS-CoV-2	Spike glycoprotein	N/A	[116]
Ebola	Viral RNA	Antiresonant reflecting optical waveguide	[117]

N/A—Not Applicable.
